# Medicine in Fiction

**Published:** 1901-02

**Authors:** 


### EDITORIAL NOTES AND COMMENTS.

The Business office of THE JOURNAL-RECORD is No. 64 Marietta Street.

The Editorial office is 318-319-320 The Prudential.

Address all Business communications to Dr. M. B. Hutchins, Mgr.

Make remittances payable to THE ATLANTA JOURNAL-RECORD OF MEDICINE.

On matters pertaining to the Editorial and Original Communications address Dr. Bernard Wolff, Atlanta.

Reprints of original articles will be furnished at cost price. Requests for the same should always be made *on the manuscript*.

We will present, post-paid, on request, to each contributor of an original article, twenty (20) marked copies of THE JOURNAL-RECORD containing such article.

### MEDICINE IN FICTION.

IT is a matter of mild surprise that fiction writers who weave into their stories the finest spun strands of astute analysis of human character and minute dissection of its motives and impulses should display such crass and absurd ignorance in their understanding of medical affairs.

In a rather clever story by a woman writer in a recent number of a high-class magazine, the bedside battle of a country doctor of the somewhat overworked McLure type, with a case of cholera infantum in a child, is described with presumed realistic fidelity to detail. We catch a glimpse of the tender thigh bared for the prick of the hypodermic needle, that later on appears as the instrument for hypodermoclysis in threatened collapse. This was followed by the ice pack despite prostration, showing the effect of Ian McLaren's tip on the Thure Brand method, but inappropriately applied. Grave misgivings would be aroused of a doctor who left his thermometer between the teeth of a sleeping child. It might be swallowed, and to revive a rusty gag, cause the child to die by degrees. Yet the doctor conquered, or rather, the patient survived, and at the end of twelve hours convalescence was assured. There are many who will weep over the tender pathos of the story, there are others who may be tempted to do so by the doctor's eccentricities of treatment.

The conception is true of human emotion, but medically viewed it is a blighted ovum.

A prime favorite among romance mongers is the house surgeon. He is liberally spread over fiction, but uniformly in a position of misunderstood function.

This individual spends his time in abetting an affaire d'amour between a privileged visitor and a pretty nurse, or is perhaps himself ensnared in her toils. When not so engaged he may be found at work in the laboratory on some obscure problem in nervous disease or "microbes." He is by preference a small man whose unobtrusive brown beard and mustache conceal a firm mouth and prognathous jaw, considered by those who possess it as indicative of great determination and resoluteness of character. The air of the hospital is one of charming domesticity and acute interest in each patient's personal and clinical history, and its administrative affairs are marked by a singular freedom from reserve. Over all this the house surgeon magisterially reigns. Those who know the larval condition of this functionary in an American hospital will appreciate how far afield in his depiction of him, the novelist has strayed.

Toxicology was once irresistibly attractive to the fictionist and the playwright. It is now somewhat out of vogue. The writer's knowledge of the subject was liable to be restricted to a certain colorless liquid of unknown composition but wonderful potency. This he used alike upon the ingenue or the villian whether for suicide or homicide, or both. Its characteristic effects upon the victim are rapidity of action, producing loss of equilibrium, convulsion, drawling hyphenated speech prolonged to but not extended beyond the point of divulgence of the missing will, the mystery of parentage, or the name of the miscreant who perpetrated the crime. The maddening hiatus remains to be filled by the hero's own efforts, the confession of the old nurse, or the dying declaration of the wounded duellist.

Chloroform is a powerful adjuvant to the success of the villian's plot. It is administered on a sponge and unconsciousness is produced in something less than a pig's whisper. The anesthetic properties of ether have not yet been utilized for fiction purposes, probably from some groping knowledge that narcosis is effected too slowly to overcome the guard or to accomplish the abduction. Aside from such general medical misapprehensions details crop up now and then in fiction which furnish hints of the profundity of the author's ignorance of technical medical matters. In describing a self-inflicted pistol shot wound of the throat, the writer of a popular novel speaks of the bullet grazing the jugular artery; another writer locates the femoral artery in the chest; another speaks of the "secum" being abnormally developed in the Esquimaux; while du Maurier's death was ascribed by one to an "abscess on the heart."

Ignorance is always rasping, but how much more harsh and obtrusive is it when set in elaborate culture! It is like a privy set in a grassy lawn, or a dish of hog-jowl and cowpeas on the banquet table of Lucullus.

The rectification of errors and misconceptions perhaps lies in the approaching age of perfection. It may produce a novelist who can deal with his medical facts and situations with the same skill that Kipling uses in mechanics, or Clarke Russell in ship lore and navigation, or Dickens in legal complications, or Balzac in finance and speculation. "The Autobiography of a Quack," was a step in the right direction, but the doctor already knows. It is the lay writer who must instruct himself, else the growing knowledge of the newspaper-reading public in medical matters will expose his deficiencies and he will get not praise, but the horse-laugh.

---

DR. GEORGE W. GOULD has been deposed by the Board of Trustees from the office of editor of the *Philadelphia Medical Journal*. The apparent cause is a difference of opinion between the editor and the business management. Dr. J. Hendrie Lloyd and Dr. Julius Salinger have been appointed to succeed Dr. Gould.

There is no more robust figure in medical journalism than Dr. Gould. Vigorous in thought and expression, fearless and remorseless in the pursuit of sham and imposture, a terror to his enemies, a beacon-light to the cause of truth and science, a man of prodigious energy, an author of note, and in the prime of his productiveness it is difficult to conceive of one more admirably suited to conduct the editorial management of a large and influential medical weekly than Dr. Gould. It is equally impossible to conceive of such a trenchant blade being permitted to rust in its scabbard, and we are not surprised to receive the information that Dr. Gould contemplates the establishment in the near future of a medical journal owned and conducted entirely within the medical guild. There can be no question about the success of such a venture, as the profession is growing more and more fretful over the increasing commercialism that is creeping over the medical press, and will welcome a weekly devoted solely to interests with an editorial pen that is "held in trust, to art, not serving shame nor lust."

There may be those who will criticise some phases of the policy of the *Philadelphia Medical Journal* in the past, but there are none who will not express regret at Dr. Gould's deposal.

